# Professional grief experience and psychological detachment of pregnant emergency nurses after the death of pediatric patients: a longitudinal mixed-methods study

**DOI:** 10.3389/fpsyg.2025.1715162

**Published:** 2026-01-26

**Authors:** Man Zhang, Dongyang Wang

**Affiliations:** 1 School of Public Health and Health Management, Henan Medical College, Zhengzhou, China; 2 Department of Nursing, The Third People’s Hospital of Henan Province, Zhengzhou, China

**Keywords:** emergency, pregnant nurses, professional grief, psychological detachment, qualitative study

## Abstract

**Background:**

Emergency nurses are frequently exposed to pediatric death events, which trigger professional grief and impair psychological detachment. Pregnant nurses, due to heightened physiological and psychological sensitivity, may exhibit unique emotional responses, yet evidence remains scarce.

**Methods:**

This study adopted a longitudinal mixed-methods design, combining semi-structured qualitative interviews with quantitative assessment using the DASS-21 scale at three time points. Data were analyzed using Colaizzi’s seven-step method to extract themes and depict temporal trajectories.

**Results:**

Five core themes were identified: (1) emotional shock—within 1 week, participants reported intense grief, anxiety, and guilt, with mean DASS-21 stress scores >15; (2) guilt and self-blame—nurses attributed death to their own limitations, prolonging recovery; (3) emotional fluctuation—by 1 month, early- and mid-pregnancy nurses showed ~30% reduction in DASS-21 scores, whereas late-pregnancy participants exhibited minimal improvement; (4) emotional support—family and peer support facilitated partial recovery; (5) physiological factors—late-pregnancy nurses maintained high anxiety (>12) and depression (>15) at 3 months, while those who had delivered showed marked emotional improvement. The overall trajectory followed shock–guilt/self-blame–impaired detachment–gradual recovery.

**Conclusion:**

Pregnancy stage markedly shapes emotional recovery after pediatric death events, with late pregnancy identified as a high-risk period. Guilt is the central barrier to psychological detachment, while social support and postpartum transitions act as protective factors. Tiered psychological interventions tailored to pregnancy stage are recommended to promote recovery and resilience among emergency nurses.

## Background

The outcomes of pediatric critical care directly affect both family and societal burden while profoundly impacting the psychological well-being of frontline healthcare workers. Globally, although the under-five mortality rate (U5MR) has shown a sustained decline, the absolute number of deaths remains considerable, with neonatal deaths accounting for a disproportionately high proportion (He et al., 2024; Sharrow et al., 2022; Perin et al., 2022). In China, for example, the most recent national surveillance data reported approximately 65,700 deaths among children under five in 2022, corresponding to a U5MR of 6.8 per 1,000 live births, with 45.1% occurring during the neonatal period. Injuries, congenital anomalies, preterm complications, and acute respiratory infections were identified as the leading causes (He et al., 2024). This epidemiological profile underscores that, across the “time–cause” dimension, pre-hospital and emergency encounters remain pivotal windows in the life trajectory of critically ill children. International estimates similarly show that, in 2019, about 5.3 million children under five died worldwide, mainly from preterm complications, lower respiratory tract infections, and birth-related asphyxia (Sharrow et al., 2022; Perin et al., 2022). Collectively, these data frame emergency nurses’ frequent, proximal exposure to pediatric deaths and the heightened emotional risks inherent in such encounters.

Within clinical settings, nurses in pediatric and general emergency departments must execute intensive resuscitation and communication tasks under severe time pressure, incomplete clinical information, and in the presence of distressed families. When resuscitation fails and a child dies, nurses are often required to immediately deliver “bad news” to families, manage postmortem procedures and equipment withdrawal, conduct case debriefing, and rapidly refocus on subsequent patients. This succession of tasks constitutes “repeated trauma exposure within a single shift” and magnifies their experience of professional grief (Kotler et al., 2024; Budak and Ay Kaatsız, 2025). Recent qualitative studies have illuminated the multifaceted challenges that pediatric and emergency staff face in end-of-life care, ethical decision-making, and emotional regulation, often reporting grief, helplessness, guilt, and moral distress. Moreover, contextual factors—such as a noisy environment, lack of privacy, and sudden deterioration—further intensify their sense of frustration (Rico-Mena et al., 2023; El-Ashry et al., 2025; Bove et al., 2025). Such experiences are rarely isolated incidents but tend instead to unfold as dynamic trajectories influenced by organizational climate and peer support.

In stress psychology and patient safety research, the Second Victim Phenomenon (SVP) provides a useful framework to understand healthcare providers’ psychological responses following adverse outcomes. Symptoms may include self-blame, fear, sleep disturbance, work avoidance, and heightened turnover intention. While peer-support interventions and structured organizational programs can offer partial relief, evidence of their long-term effectiveness remains limited (Ong et al., 2025). Systematic reviews suggest that tiered peer-support models yield short-term emotional benefits; however, their impact on burnout, retention, and other long-term outcomes remains inconclusive due to heterogeneity in measurement tools and follow-up timelines (Sedile et al., 2024; Copley et al., 2024; Alishaq et al., 2025). In cases of non-preventable, unexpected failures in resuscitation (e.g., pre-hospital cardiac arrest, trauma, or septic shock), little is known about whether nurses’ grief responses follow SVP-like phases, or how these responses hinder subsequent “psychological detachment” during rest periods (Finney et al., 2021).

Psychological detachment—a central construct in recovery theory—refers to an individual’s ability to mentally disengage from work-related thoughts and emotions during non-work hours (Sagherian et al., 2023; Zhang et al., 2023). Evidence shows significant associations between detachment and sleep quality, mental health, and burnout (Cho et al., 2025). Empirical studies and meta-analyses indicate that effective detachment is linked to reduced emotional exhaustion, greater well-being, and lower turnover intention, including among nurses (Blake et al., 2025; Karabinski et al., 2021). However, most existing studies are cross-sectional or measure detachment at a single time point, focusing on general stressors such as workload, incivility, or team support. Few have examined how event-specific traumatic grief (e.g., following a child’s death) disrupts detachment, or whether such interference diminishes or recurs over time. This gap is especially pronounced for pregnant nurses.

Pregnant nurses represent a particularly vulnerable group, with heightened physiological sensitivity (e.g., hormonal fluctuations, sleep alterations, physical load) and psychological sensitivity (e.g., perceived risks to fetal safety). Epidemiological and occupational health studies have increasingly associated night shifts and rotating shifts during pregnancy with adverse outcomes such as preterm birth and small-for-gestational-age infants, although findings vary across studies (Kader et al., 2022; Lee et al., 2024; Adane et al., 2023; Kowalenko et al., 2024). Broader evidence also links psychosocial stress and workload during pregnancy to increased risk of obstetric complications (Hino et al., 2025). In emergency departments, pregnant nurses are simultaneously exposed to intense shift work demands and the emotional toll of pediatric deaths, rendering them prone to cycles of “self-protection—emotional withdrawal—guilt resurgence.” Post-shift difficulties in “switching off,” intrusive recollections of failed resuscitation, and poor sleep quality may all manifest as impaired psychological detachment. Such cycles—grief, impaired detachment, fatigue/sleep disturbance, and heightened emotional vulnerability in subsequent shifts—risk undermining both work engagement and patient safety. Although some hospitals have introduced peer support, structured debriefings, and digital mental health resources, these measures rarely target pregnant nurses or adopt longitudinal designs capable of mapping recovery trajectories at 1-week, 1-month, and 3-month intervals.

Further, studies in Chinese nursing populations suggest that team support and work–nonwork boundary management are strongly associated with detachment, burnout, and turnover intention (Karabinski et al., 2021). Yet, few investigations have examined major adverse outcomes as specific triggers, and even fewer have incorporated pregnancy as a stratification variable. Cultural factors may also shape responses: norms of grief expression and mourning rituals in Chinese contexts likely influence nurses’ communication, emotional processing, and coping strategies, thereby affecting detachment and recovery. Considering the rising proportion of injury-related child deaths in China, persistent urban–rural disparities (He et al., 2024), and the accelerated pace of emergency care delivery, there is urgent need for temporally nuanced evidence on “pregnant emergency nurses—grief after pediatric death—psychological detachment.” Such research holds both managerial and occupational health relevance and could inform evidence-based “post-event support” programs and work-scheduling strategies.

In summary, pediatric deaths are not uncommon in emergency care and expose nurses to frequent, proximal emotional and ethical challenges. The frameworks of professional grief and SVP help explain the multidimensional responses of healthcare providers, yet research on pregnant nurses with temporal sensitivity remains scarce. Psychological detachment is closely tied to nurses’ sleep, emotional health, burnout, and retention, but the causal chain linking event-specific grief and impaired detachment remains underexplored. Therefore, this study adopts a convergent longitudinal mixed-methods design with a descriptive phenomenological qualitative core to map the evolving trajectory of professional grief among pregnant emergency nurses at 1-week, 1-month, and 3-month intervals following pediatric deaths. It further examines how these grief experiences influence psychological detachment (content, intensity, and rhythm) during non-work time and their subsequent impact on work and life domains, thereby providing context-specific, actionable evidence to guide tailored post-event support, recovery pathways, and scheduling strategies for pregnant nurses.

## Methods

### Study design

This study employed a Convergent Longitudinal Mixed Methods Design, grounded in the Pragmatist paradigm. Pragmatism focuses on “what works” to answer research questions, rejecting the forced choice between post-positivism and constructivism. In this study, we collected both quantitative (DASS-21) and qualitative (interview) data concurrently at three time points (1 week, 1 month, 3 months). The mixed-methods approach allowed the study to capture both the depth of personal experience and the measurable progression of psychological symptoms over time, thereby directly addressing the multidimensional nature of grief, guilt, and work-related detachment. The qualitative component was grounded in descriptive phenomenology to explore nurses’ lived experiences, subjective interpretations, and evolving emotional meanings. In parallel, the quantitative component utilized the DASS-21 scale at three intervals to monitor changes in depression, anxiety, and stress symptoms.

This study adopted a Husserlian descriptive phenomenological orientation, operationalized through Colaizzi’s method of analysis in qualitative parts. The longitudinal design was informed by prior international phenomenological studies that explore the temporal unfolding of lived experience, recognizing that some phenomena cannot be fully understood at a single time point. Longitudinal phenomenological inquiry has been employed in studies examining grief trajectories, evolving self-understanding, and shifting embodied experiences across critical life phases. In this tradition, repeated interviews do not aim to alter the phenomenon but rather to capture its temporal structure—that is, how meaning is constituted, stabilized, or transformed over time. Accordingly, the three interview points in this study were not intended to compare participants against themselves as “data points,” but to access different layers of the lived experience as participants revisited and reinterpreted the event across pregnancy stages.

### Philosophical framework

This study was grounded in Husserlian descriptive phenomenology, which seeks to describe the essence of lived experience through intentionality, reduction, and the identification of meaning structures. Colaizzi’s method was selected because it operationalizes Husserl’s principles by systematically transforming significant statements into formulated meanings and synthesized themes. Phenomenological reduction was maintained through bracketing, reflexive journaling, and iterative return to participants’ verbatim descriptions.

### Participants and recruitment

The study was conducted in a large tertiary general hospital located in Zhengzhou, Henan Province, China. The hospital has approximately 1,800 beds and employs more than 900 nurses, serving as one of the major referral centers in central China. Each year, the emergency department receives more than 300,000 visits, including a high volume of pediatric emergencies. The study was conducted in the emergency department (ED) of this hospital, an environment characterized by high patient acuity, rapid clinical turnover, and frequent exposure to traumatic pediatric cases.

The sampling frame consisted of pregnant nurses working in ED who had directly participated in pediatric resuscitation attempts that resulted in patient death within the previous 12 months. Eligible nurses were identified through the emergency department’s standardized resuscitation records and death documentation logs, ensuring that all participants experienced real, verifiable exposure to the type of critical event under investigation. The emergency department head nurse assisted in verifying individual involvement in specific pediatric death events to ensure sampling accuracy.

Inclusion criteria required that participants: (1) were pregnant female nurses working in the ED during pregnancy; (2) had personally participated in pediatric resuscitation and directly experienced a child’s death; (3) possessed sufficient communication ability to clearly articulate their emotions and psychological states; and (4) voluntarily agreed to participate in at least three interviews at 1 week, 1 month, and 3 months post-event.

Exclusion criteria included: (1) severe pregnancy-related complications (e.g., gestational hypertension, intrauterine growth restriction, gestational diabetes) that might influence emotional responses; (2) severe psychiatric disorders such as major depression, anxiety disorder, or post-traumatic stress disorder (PTSD); (3) inability to attend at least two interviews.

### Sampling and recruitment

A purposive sampling strategy was used initially to recruit nurses from the defined sampling frame, ensuring variability in pregnancy stage, years of clinical experience, professional rank, and shift type (day vs. night). Snowball sampling was subsequently used to recruit additional participants who met the same eligibility criteria. Recruitment continued until thematic saturation was reached, which occurred when no new meaning units, concepts, or experiential variations emerged during iterative transcript analysis. A total of 18 pregnant emergency nurses participated in the study. All participants completed informed consent prior to enrollment.

### Data collection timeline and procedures

Data collection occurred between June 2024 and May 2025. Each participant completed three qualitative interviews and three DASS-21 assessments at approximately 1 week, 1 month, and 3 months following the pediatric death event. These intervals were selected based on grief and trauma literature that emphasizes acute, intermediate, and sustained phases of response, and to ensure sufficient temporal sensitivity in the mixed-methods design.

Interviews were conducted in a private consultation room located within the emergency department, or via encrypted videoconferencing when participants required additional privacy or experienced pregnancy-related physical limitations. Each interview lasted between 60 and 90 min. All interviews were audio-recorded with participant permission and transcribed verbatim by trained research assistants. Transcripts were reviewed for accuracy by two independent researchers, and all identifiable information was removed to protect confidentiality.

### Qualitative data collection

A single semi-structured interview guide was used consistently across all three timepoints to ensure methodological comparability, while time-specific probes were added to explore emotional changes relative to previous interviews. The interview guide included domains related to recollection of the pediatric death event, grief reactions, guilt, intrusive memories, psychological detachment during non-work hours, coping strategies, support systems, pregnancy-related physiological changes, and perceived barriers to emotional recovery.

### Quantitative component: DASS-21 assessment

The Depression Anxiety Stress Scales-21 (DASS-21) was employed to quantitatively assess emotional distress at all three timepoints. The DASS-21 is a well-validated psychological instrument developed by Lovibond and Lovibond, consisting of 21 items divided into three subscales that measure depression, anxiety, and stress. Items were rated on a four-point Likert scale ranging from zero (“did not apply to me at all”) to three (“applied to me most of the time”). Subscale scores were summed and multiplied by two to align with the DASS-42 scoring framework. Severity classifications followed established cut-off thresholds for each subscale. The Chinese-language version of the DASS-21, which has shown strong psychometric reliability in healthcare populations, was used in this study. Internal consistency in our sample was high (Cronbach’s *α* = 0.86), indicating suitability for repeated emotional assessments among pregnant nurses.

### Qualitative data analysis

Qualitative data were analyzed using Colaizzi’s seven-step phenomenological method. All transcripts were read repeatedly to obtain a holistic understanding of the emotional and cognitive processes described by participants. Significant statements relevant to grief responses, psychological detachment, work-related stress, pregnancy-related vulnerability, and perceived barriers or facilitators of recovery were extracted and transformed into formulated meanings. Meaning units were then clustered into subthemes, which were subsequently synthesized into major themes representing higher-order experiential constructs.

To ensure rigor, two independent coders analyzed the transcripts and compared coding structures. Discrepancies were resolved through iterative discussion. Member checking was conducted by returning thematic summaries to participants to confirm accuracy and interpretive fidelity. Both cross-sectional and longitudinal analyses were conducted to identify emotional differences between participants at each timepoint and within each participant across time.

### Quantitative data analysis

Descriptive statistics were used to summarize DASS-21 scores at all three timepoints. Due to the small sample size (n = 18), inferential statistical testing was not performed. Data were analyzed using descriptive statistics (means, trends) to identify patterns over time. Means, standard deviations, and score ranges were calculated for depression, anxiety, and stress subscales.

### Reflexivity statement

The researchers engaged in continuous reflexive practice throughout the study to ensure that the findings remained grounded in participants’ lived experiences rather than researchers’ assumptions. The interviews were conducted by two female researchers trained in qualitative and phenomenological methods who were not part of the hospital workforce and had no supervisory or clinical relationships with the participants. Prior to data collection, the researchers documented potential preconceptions related to grief, pregnancy, and emergency nursing and bracketed these assumptions through reflexive journaling. Peer debriefing sessions were held during data analysis to discuss interpretive decisions, challenge emerging assumptions, and enhance analytic rigor. These strategies helped ensure that the interpretation remained closely aligned with participants’ narratives and minimized the influence of researcher bias.

### Ethical considerations

Ethical approval was obtained from the Institutional Review Board of the participating hospital. Written informed consent was obtained from all participants prior to data collection. Because the study involved emotionally sensitive content, interviewers were trained to monitor participant distress and to pause interviews when necessary. Participants were offered access to psychological counseling services if needed. All electronic data were stored in encrypted files with restricted access to maintain confidentiality and data security.

## Results

### Participant characteristics

A total of 18 emergency nurses were recruited, all of whom were female. Participants ranged in age from 28 to 35 years, with a mean age of 31.25 ± 2.33 years. Their years of work experience ranged from 2 to 8 years, with an average of 4.88 ± 2.25 years. Educational attainment was generally high, with most holding a bachelor’s degree or higher; six were senior nurses or nurse-in-charge, and two were registered nurses. Participants were distributed across early, middle, and late stages of pregnancy (Table 1). In terms of shift schedules, 15 participants primarily worked day shifts, while three participants (P4, P10, and P16) were on night duty when the event occurred. Night-shift nurses exhibited higher levels of emotional distress than day-shift nurses, as reflected by elevated DASS-21 scores and slower recovery trajectories. Across one-week to three-month follow-ups, night-shift nurses consistently reported more pronounced symptoms of anxiety and depression, suggesting that shift work and circadian rhythm disruption may exacerbate the impact of professional grief. Emotional recovery also varied by pregnancy stage: early- and mid-pregnancy participants generally showed smoother recovery, whereas late-pregnancy participants recovered more slowly. Some in late pregnancy had delivered by the three-month follow-up, and the physiological transition of childbirth interfered with the recovery process.

**Table 1:** Basic characteristics of participants.

**Participant ID**	**Years of work**	**Education**	**Gestational stage**	**Remarks**
P1	5	Bachelor	Mid-pregnancy	Gradual emotional relief, good recovery
P2	3	Bachelor	Early pregnancy	Rapid recovery, emotional fluctuations due to pregnancy
P3	8	Master	Late pregnancy	Mild fluctuations, faster recovery
P4	2	Diploma	Mid-pregnancy	Slow recovery, large fluctuations
P5	4	Bachelor	Late pregnancy	Pregnancy factors influenced emotions, incomplete recovery
P6	5	Bachelor	Early pregnancy	Faster recovery
P7	6	Bachelor	Mid-pregnancy	Good recovery
P8	7	Master	Late pregnancy	Stable emotions, good recovery
P9	6	Master	Mid-pregnancy	Mild fluctuations, rapid recovery
P10	3	Diploma	Mid-pregnancy	Large fluctuations, incomplete recovery
P11	4	Bachelor	Late pregnancy	Strong fluctuations, needs more support

### Core coding features

Systematic analysis of the interview data identified several Themes reflecting the emotional responses and recovery trajectories of pregnant emergency nurses. Emotional shock was a universal initial response following pediatric death. Participants reported intense distress, manifested as severe anxiety, grief, guilt, and emotional loss of control, making it difficult to cope with overwhelming emotional fluctuations (Table 2). Guilt and self-blame were pervasive, as many participants felt they had failed the child and internalized responsibility for the death. These negative emotions hindered emotional recovery, with some unable to overcome self-blame in the short term, which further impaired work performance and emotional regulation. Emotional volatility and confusion were also common, with participants describing unstable patterns, alternating between partial recovery and emotional relapse. This phase was marked by confusion, unease, and slow progress in emotional stabilization. Over time, emotional support and self-regulation contributed to gradual recovery, as effective involvement of support systems and personal coping strategies helped participants regain stability. Pregnancy-related fluctuations emerged as a critical factor, particularly in late pregnancy. Physiological changes such as hormonal shifts and fetal health concerns amplified distress, and some late-pregnancy participants showed persistently high DASS-21 scores, underscoring the compounding effect of pregnancy on grief recovery.

**Table 2:** Emotional trajectory of participants.

Time point	Themes	Subthemes
1 week	Initial emotional shock	Intense grief, anxiety, guilt, with pronounced instability and difficulty adapting
	Guilt and self-blame	Strong sense of responsibility for the child’s death, emotional suppression, difficulty in expression
	Emotional fluctuation and confusion	Persistent instability, alternating between recovery and relapse
	Initial psychological detachment	Difficulty concentrating, frequent intrusive memories of the death, impaired work performance
1 month	Emotional fluctuation and recovery	Gradual relief, but ongoing distress; recovery is nonlinear with persistent grief and guilt
	Adapting to work roles	Adjusting schedules and strategies to cope
	Emotional regulation and support	Seeking social/peer support and using self-regulation
	Emotional expression	Difficulty expressing emotions, especially in professional settings
3 months	Emotional recovery and balance	Emotions gradually stabilized, though some anxiety persisted; recovery varied
	Role of emotional support	Family and peer support played a critical role in easing distress
	Maturation of regulation	Nurses developed self-regulation and acceptance of the event

### DASS-21 quantitative findings

Descriptive statistics were calculated for depression, anxiety, and stress scores at 1 week, 1 month, and 3 months after the event. Mean scores across participants showed a consistent decline over time, indicating progressive emotional recovery. At 1 week, mean scores were highest across all three subscales (Depression = 15.83; Anxiety = 13.50; Stress = 17.44), with moderate variability (SDs 2.79–3.11). By 1 month, average scores had decreased substantially (Depression = 13.17; Anxiety = 10.78; Stress = 13.56), and continued to decline at 3 months (Depression = 11.06; Anxiety = 9.06; Stress = 11.22), reflecting broad improvement in psychological distress levels (Table 3).

**Table 3:** DASS-21 score of participants.

Participant ID	DASS-21 scores (1 week)	DASS-21 scores (1 month)	DASS-21 scores (3 months)
P1	Dep 13, Anx 10, Str 15	Dep 8, Anx 5, Str 9	Dep 6, Anx 4, Str 8
P2	Dep 18, Anx 16, Str 20	Dep 12, Anx 10, Str 14	Dep 10, Anx 7, Str 10
P3	Dep 14, Anx 12, Str 15	Dep 12, Anx 9, Str 11	Dep 9, Anx 7, Str 8
P4	Dep 20, Anx 18, Str 22	Dep 18, Anx 16, Str 19	Dep 15, Anx 13, Str 17
P5	Dep 19, Anx 16, Str 21	Dep 17, Anx 14, Str 18	Dep 16, Anx 13, Str 17
P6	Dep 14, Anx 12, Str 15	Dep 10, Anx 8, Str 10	Dep 8, Anx 6, Str 8
P7	Dep 15, Anx 13, Str 16	Dep 13, Anx 11, Str 14	Dep 10, Anx 8, Str 9
P8	Dep 13, Anx 11, Str 14	Dep 11, Anx 9, Str 10	Dep 9, Anx 7, Str 8
P9	Dep 14, Anx 12, Str 15	Dep 11, Anx 9, Str 12	Dep 8, Anx 7, Str 9
P10	Dep 22, Anx 18, Str 23	Dep 20, Anx 17, Str 20	Dep 17, Anx 15, Str 18
P11	Dep 18, Anx 16, Str 19	Dep 16, Anx 14, Str 18	Dep 15, Anx 13, Str 16
P12	Dep 13, Anx 11, Str 15	Dep 10, Anx 8, Str 10	Dep 7, Anx 6, Str 7
P13	Dep 14, Anx 12, Str 16	Dep 12, Anx 10, Str 13	Dep 10, Anx 8, Str 9
P14	Dep 20, Anx 17, Str 22	Dep 18, Anx 15, Str 18	Dep 16, Anx 14, Str 16
P15	Dep 12, Anx 10, Str 14	Dep 9, Anx 7, Str 9	Dep 8, Anx 6, Str 8
P16	Dep 18, Anx 15, Str 19	Dep 16, Anx 13, Str 16	Dep 14, Anx 12, Str 14
P17	Dep 16, Anx 14, Str 18	Dep 14, Anx 12, Str 14	Dep 13, Anx 11, Str 12
P18	Dep 12, Anx 10, Str 15	Dep 10, Anx 7, Str 9	Dep 8, Anx 6, Str 8

Dep: Depression; Anx: Anxiety; Str: Stress.

Temporal trends were evident across subgroups. Pregnancy stage showed distinct recovery patterns: early-pregnancy nurses generally exhibited faster score reductions, particularly in anxiety and stress, whereas late-pregnancy participants demonstrated more persistent elevations and slower decline. These differences aligned with qualitative reports of heightened physiological burden and anticipatory anxiety in late-stage pregnancy. Similarly, shift patterns produced clear contrasts: day-shift nurses showed steady improvement across all subscales, while night-shift nurses—particularly P4, P10, and P16—displayed higher baseline distress and more gradual declines over time. These subgroup differences added interpretive depth to the descriptive results and corresponded closely with qualitative themes describing fatigue, disrupted sleep cycles, and limited social support among night-shift participants (Table 4).

**Table 4:** DASS-21 scores by gestational stage × work shift.

Gestational stage	Shift	Dep 1W	Dep 1M	Dep 3M	Anx 1W	Anx 1M	Anx 3M	Str 1W	Str 1M	Str 3M
Early pregnancy	Day shift	15.00	10.67	8.33	13.00	8.67	6.33	16.67	11.33	8.33
	Night shift	18.00	16.00	14.00	15.00	13.00	12.00	19.00	16.00	14.00
Mid-pregnancy	Day shift	13.33	10.50	8.33	11.17	8.17	6.50	15.17	11.00	8.50
	Night shift	21.00	19.00	16.00	18.00	16.50	14.00	22.50	19.50	17.50
Late pregnancy	Day shift	16.67	14.67	13.00	14.33	12.17	10.83	18.17	14.83	12.83
	Night shift	—	—	—	—	—	—	—	—	—

### One-week follow-up

During the first week, all participants experienced strong emotional shock, severe mood swings, and difficulty regaining balance. Emotional responses were dominated by grief, anxiety, and guilt, with widespread self-blame for being unable to save the child (Table 5). For instance, P1 (mid-pregnancy, 5 years of experience) scored 13 for depression, 10 for anxiety, and 15 for stress, and reported: “When I saw the child’s face, my world suddenly collapsed. It felt like everything had stopped.” Night-shift nurses demonstrated particularly intense distress. P4 (mid-pregnancy, night shift) scored 22 for stress, higher than most day-shift counterparts, describing isolation and fatigue as amplifiers of her grief. P10 (mid-pregnancy, night shift) scored 18 for anxiety and 23 for stress, stating: “I could not sleep for a long time, the failed resuscitation kept replaying in my mind.” P16 (early pregnancy, night shift) also reported severe insomnia and intrusive recollections, with a depression score of 18, markedly higher than day-shift peers. Although day-shift nurses also experienced strong emotional shock, some demonstrated partial buffering effects through family support and peer communication. For example, P2 (early pregnancy, day shift) scored 16 for anxiety, but reported: “Through communicating with my partner, I was able to release some of my emotions.” Overall, the defining features of the first week were “emotional shock—peaks of self-blame and anxiety—marked impairment in psychological detachment,” with night-shift nurses displaying higher intensity and persistence of distress compared to their day-shift counterparts.

**Table 5:** Themes, subthemes, and participants’ quotations (within 1 week).

**Theme**	**Subtheme**	**Participant quotation**
Initial emotional shock	Intense emotional impact	“When I saw the child’s face, my world suddenly collapsed. I felt like everything had stopped.” (P9)
	Heavy psychological burden	“I kept asking myself why I did not act faster or do more. I felt like a failure.” (P3)
Guilt and Self-Blame	Self-blame	“I wonder if the child might still be alive if I had done better.” (P2)
	Sense of responsibility toward family and patients	“I feel I let down both my family and the patient’s parents, as if I did not do enough.” (P6)
Emotional fluctuation and confusion	Extreme mood swings	“Sometimes I burst into tears, sometimes I go numb. I do not even know what I’m feeling.” (P10)
	Psychological confusion	“My mind goes blank, my heart races, I’m terrified of failing to save another child.” (P4)
Initial psychological detachment	Difficulty focusing at work	“I could not concentrate all day. Each time I saw a child, I relived that moment.” (P12)
	Inner helplessness	“I felt empty inside, often powerless facing patients and families.” (P8)

### One-month follow-up

By the second stage (within 1 month), some participants showed gradual emotional recovery, with DASS-21 scores generally trending downward, indicating improvement. Early- and mid-pregnancy participants, in particular, demonstrated smoother recovery (Table 6). For example, P6 (early pregnancy, 5 years of experience) reported DASS scores of 10 for depression, 8 for anxiety, and 10 for stress at 1 month. She stated: “Although my emotions have somewhat recovered, I still often recall that event,” reflecting partial recovery while continuing to experience lingering impact. Similarly, P8 (mid-pregnancy, 6 years of experience) reported reductions in her DASS scores (depression 9, anxiety 6, stress 7), explaining: “Through communication with my family and my own adjustments, my emotions have improved.” These cases illustrate the positive role of support systems in alleviating distress and stabilizing emotions during this phase.

**Table 6:** Themes, subthemes, and participants’ quotations (within 1 month).

Theme	Subtheme	Participant quotation
Emotional fluctuation and recovery	Gradual relief	“A month has passed, and I do not feel as painful as before, but there is still a knot in my heart. I often think of that child.” (P5)
	Ongoing fluctuations	“Although a month has gone by, I sometimes fall back into sadness, especially when recalling that day.” (P14)
Adapting to work roles	Role adjustment	“I try to schedule more rest and avoid taking on overly heavy tasks.” (P13)
	Self-protection strategies	“I try to control my emotions and avoid too much emotional engagement with families. That helps me cope.” (P7)
Emotional regulation and support	Seeking support	“I talked to my family many times. Even though they do not fully understand, their support made me feel better.” (P11)
	Self-regulation	“I go jogging, meditate—these make me feel calmer and help me let go of some emotions.” (P6)
Emotional expression	Expression and inhibition	“At work I try to stay calm, but after repressing my emotions all day, I sometimes cry alone at night.” (P10)

However, recovery was slower among late-pregnancy participants. For instance, P5 (late pregnancy, 4 years of experience) still exhibited elevated DASS scores 1 month post-event (depression 15, anxiety 12, stress 14). She reported: “Emotional fluctuations are very intense in late pregnancy, and I cannot calm my anxiety, which is largely related to physiological changes during pregnancy.” These findings highlight how pregnancy-related factors, such as hormonal fluctuations and fetal health concerns, hinder emotional recovery. Night-shift nurses also demonstrated noticeably delayed recovery compared to day-shift nurses. For example, P10’s depression and stress scores remained at 20 after 1 month, only slightly improved from the first week. P4 and P16 also maintained high levels of distress, both reporting that “emotional fluctuations are stronger when alone at night.” These narratives indicate a persistent state of impaired psychological detachment, where the solitude of night shifts prevents the mental disengagement necessary for recovery.

### Three-month follow-up

By the third stage (within 3 months), most participants demonstrated notable emotional recovery, with DASS-21 scores showing further reductions and emotions trending toward stability (Table 7). For instance, P12 (mid-pregnancy, 6 years of experience) reported DASS scores of 5 for depression, 4 for anxiety, and 6 for stress. She stated: “My emotions gradually recovered, the fetal health issues were alleviated, and I felt much more relaxed.” This suggests that both physiological stability and emotional support contributed to recovery.

**Table 7:** Themes, subthemes, and participants’ quotations (within 3 months).

Theme	Subtheme	Participant quotation
Emotional recovery and balance	Gradual recovery	“Although I feel much better now, whenever I see similar cases, I still feel nervous, afraid it will happen again.” (P12)
	Long-term fluctuation	“Three months later, my emotions are much more stable, but I still occasionally feel anxious and unsettled.” (P16)
Role of emotional support	Family and colleague support	“My family has always supported me. Even if they do not fully understand, their presence gives me strength.” (P9)
	Diverse support sources	“I talk with my colleagues—they understand me and do not think I’m being overly sensitive. We support each other.” (P4)
Maturation of emotional regulation	Self-regulation strategies	“Now I can accept the experience, using meditation and deep breathing to regulate my emotions.” (P8)
	From grief to acceptance	“Although I have not completely recovered, I’ve gradually learned to accept it and not let myself stay trapped in sadness.” (P15)

Nevertheless, some night-shift nurses continued to exhibit significant emotional distress at 3 months. P16 (early pregnancy, night shift) scored 14 for depression, 12 for anxiety, and 14 for stress. Although these scores decreased from baseline, her recovery remained incomplete. She noted: “I still suffer frequent insomnia at night, and my emotions fluctuate repeatedly,” indicating that the negative effect of night-shift experiences continued to manifest as a failure of psychological detachment even after 3 months. Likewise, some late-pregnancy participants continued to show slower recovery. For example, P3 (late pregnancy, 8 years of experience) recorded DASS scores of 19 for depression, 17 for anxiety, and 19 for stress. She reported: “Emotional fluctuations in late pregnancy are intense, and my anxiety has not fully subsided, especially because of excessive worries about fetal health.” Participants described this fluctuation specifically as impaired psychological detachment. They reported an inability to mentally disengage from the resuscitation scene once they returned home, leading to intrusive thoughts during rest periods. For late-pregnancy nurses, physiological discomfort (e.g., fetal movement) served as a constant somatic trigger, preventing effective psychological detachment and keeping them in a state of hyper-arousal related to the potential death of their own child. These results underscore the compounding influence of late-pregnancy physiological factors, such as hormonal changes and fetal concerns, in impeding recovery.

Interestingly, some late-pregnancy participants who had delivered by the three-month mark reported improved emotional adjustment. For instance, P18 explained: “After my baby was born, my emotions became a little better. Even though the anxiety did not disappear completely, the healthy birth of my child made me feel more relieved.” This physiological transition provided a turning point in her emotional trajectory, where the safe delivery of the infant significantly reduced anxiety and promoted emotional stability.

### Integration of findings: meta-inferences

By integrating the quantitative trajectories with qualitative themes via a Joint Display (Table 8), several meta-inferences emerged that would not have been visible using a single method alone.

**Table 8:** Joint display of quantitative scores and qualitative themes with meta-inferences.

Time point	Quantitative results (DASS-21 trends)	Qualitative themes and exemplar quotes	Meta-inference (Integrated Interpretation)
1 week	High Severity: Mean Stress score 17.44; Depression 15.83. Minimal variance between groups.	Theme: Initial Emotional Shock. *“My world suddenly collapsed. everything stopped.” (P1)*	Convergence: The acute phase is characterized by a universal, high-intensity trauma response where physiological stress markers align with cognitive paralysis, regardless of pregnancy stage.
Within 1 month	Diverging Trajectories: Early/Mid-pregnancy scores drop (~30%). Late-pregnancy & Night-shift scores remain elevated (e.g., P10 Stress: 20).	Theme: Emotional Fluctuation. *Explaining the lag: “Emotional fluctuations are stronger when alone at night.” (P4)*	Expansion: Recovery is non-linear. “Night shift” and “Late Pregnancy” act as structural and biological moderators. The statistical “failure to recover” is explained by the lived experience of isolation and hormonal burden.
3 months	Stabilization vs. Persistence: Most participants return to mild levels. Night-shift nurses (P16) retain moderate anxiety (Score: 12).	Theme: Impaired Detachment. *Context: “I still suffer frequent insomnia. emotions fluctuate repeatedly.” (P16)*	Complementarity: Statistical “residual symptoms” are qualitatively revealed as “impaired psychological detachment.” The numbers quantify the symptom, while the narrative identifies the mechanism (sleep disruption/rumination) requiring intervention.

Convergence of Acute Shock (Week 1): Quantitative data showed universally high stress scores (>15), which converged with the qualitative theme of “Emotional Shock.” The meta-inference here is that the initial impact of pediatric death is a systemic trauma response that overrides individual differences in experience or pregnancy stage.

Divergence and Explanation (Month 1 and 3): A key divergence appeared in the recovery phase. While the overall mean DASS-21 scores declined, the qualitative data explained why outliers existed. The statistical “lag” in recovery for night-shift and late-pregnancy nurses was directly explained by the qualitative subthemes of “isolation at night” and “physiological burden/fetal anxiety.” The quantitative data identified who was not recovering (late pregnancy/night shift), while the qualitative data revealed the mechanism (structural isolation and hormonal amplification).

The “Turning Point” Mechanism: The quantitative drop in anxiety scores for P18 at Month 3 seemed abrupt statistically but was contextualized by her narrative of “childbirth.” This integration suggests that for pregnant nurses, “delivery” acts as a critical biological release valve for professional grief, a finding unique to this mixed-methods inquiry.

## Discussion

### Emotional fluctuations among pregnant emergency nurses

This study found that pregnant emergency nurses exhibited significant emotional fluctuations after experiencing pediatric death events. Their reactions included not only short-term shock, grief, and anxiety but also sustained guilt and depressive symptoms. Unlike previous studies showing that “general emergency nurses often recover gradually within weeks after such events” (Hino et al., 2025), pregnant nurses in this study demonstrated delayed emotional recovery. This difference was frequently described by participants themselves in relation to the unique physiological and psychological states of pregnancy. Research has shown that hormonal fluctuations—particularly in estrogen and progesterone—have been associated with greater emotional instability and anxiety (Clayborne et al., 2022). In this study, late-pregnancy nurses were especially affected, remaining in emotional distress weeks after the event, which contrasts with the recovery trajectory observed in non-pregnant nurses or the general population. Moreover, early- and mid-pregnancy participants showed relatively faster recovery, with some nearing baseline psychological levels within a month. This differs from Chen et al., who reported significant distress among mid-pregnancy nurses (Zhang et al., 2022). The discrepancy may be due to differences in clinical departments and the type of stressors. The emergency department’s unpredictability, high intensity, and high mortality likely amplify the psychological impact of such events, thereby accentuating stage-specific differences across pregnancy.

### Unique challenges for late-pregnancy nurses

A notable finding of this study is that late-pregnancy nurses experienced the slowest recovery. Previous research has shown that women in late pregnancy are more likely to present with persistent anxiety and depressive symptoms in the context of increased physiological burden, poor sleep quality, and concerns about childbirth (Kelly and Thompson, 2022). Our findings resonate with this view, as late-pregnancy emergency nurses in this study described themselves as especially vulnerable to ongoing distress after pediatric deaths. While general nurses often recover within 2–3 months (Lee et al., 2021), late-pregnancy nurses continued to report high levels of anxiety and guilt even at 3 months. This suggests that late pregnancy constitutes a high-risk period requiring targeted psychological intervention. Interestingly, some late-pregnancy participants who had entered the postpartum stage by the three-month follow-up showed notable emotional improvement. This aligns with White et al. (2023), who found that childbirth can alleviate prenatal anxiety and promote recovery through mother–infant bonding. Thus, the “risk window” in late pregnancy may give way to a turning point after delivery, providing critical insights for timing nursing support strategies.

### Risks and recovery challenges of night-shift nurses

Another important finding is that night-shift nurses experienced more intense emotional impact and significantly delayed recovery following pediatric death events. This highlights night-shift work as an independent negative factor in professional grief among pregnant nurses, warranting close attention from clinical and managerial stakeholders. During acute-phase interviews, night-shift nurses consistently reported higher levels of anxiety and stress. Their DASS-21 stress scores were among the highest, and narratives frequently included feelings of isolation and sleep disturbance after night resuscitations. The night-shift environment deprived them of timely emotional support and effective coping resources, intensifying trauma. Previous research similarly indicated that circadian disruption and poor sleep quality make night-shift nurses more vulnerable to anxiety and depression (Kader et al., 2022). Lee et al. further confirmed that night shifts during pregnancy significantly increase risks of insomnia and psychological distress (Lee et al., 2024), findings that align closely with our qualitative data. Compared to day-shift nurses, night-shift nurses reported stronger isolation and rumination after acute stress, making short-term emotional buffering more difficult.

Across follow-ups, night-shift nurses’ recovery trajectory was markedly delayed and incomplete. From 1–3 months, their emotional improvements lagged behind those of day-shift nurses, with some still reporting moderate anxiety and depression at 3 months. These results echo previous findings on the long-term adverse effects of night-shift work during pregnancy. Adane et al. found that pregnant women working night shifts face increased risks of adverse pregnancy outcomes as well as higher rates of prenatal anxiety and depression compared with non-night-shift peers (Adane et al., 2023). Kader et al. (2022) also reported a stable association between rotating shifts and depressive risk during pregnancy, particularly with consecutive night shifts. Taken together, these findings suggest that night-shift work is experienced not only as intensifying acute distress but also as hampering longer-term recovery among pregnant nurses. Participants often described the combination of night-shift work and the physiological vulnerabilities of pregnancy as a “double burden.” Existing studies indicate that hormonal fluctuations and fetal-related anxiety are associated with challenges in emotional regulation (Hino et al., 2025; Clayborne et al., 2022; Zhang et al., 2022), while circadian disruption, reduced melatonin secretion, and sleep deprivation during night work are linked with diminished psychological resilience (Kader et al., 2022). In our study, this dual context was lived by night-shift pregnant nurses as a persistent strain. Viewing this through the lens of the stressor-detachment model (Sonnentag and Fritz, 2015), the physiological demand of night work acts as a continuous stressor that depletes the cognitive resources required to psychologically detach, thereby stalling the recovery process identified in our trajectory data.

Although social support played a generally positive role in recovery for most pregnant nurses (Sagherian et al., 2023), its effects were relatively limited for night-shift nurses. Interviews revealed frequent mentions of “no one to talk to at night” or “emotions harder to release after night duty.” Prior research shows that misalignment between night work and social support systems contributes to social isolation, reducing the protective role of support (Cho et al., 2025). Therefore, relying solely on family or peer support may not be sufficient for night-shift pregnant nurses. Additional structured interventions—such as night-shift-specific psychological support programs, peer support groups, or online counseling services—are urgently needed to bridge these gaps.

### Persistent role of guilt and self-blame

This study further revealed that guilt and self-blame significantly impeded emotional recovery among pregnant emergency nurses. Participants commonly attributed pediatric deaths to their perceived failure to act adequately, which triggered strong self-blame. This aligns with the “Second Victim Phenomenon,” which describes healthcare providers’ feelings of self-blame, helplessness, and burnout after patient death or adverse events (McCauley and Bailey, 2023). However, our study found that guilt among pregnant nurses was not only more intense but also more prolonged. This differs from McCauley et al., who observed that most healthcare workers’ self-blame decreased significantly within 6 weeks (Jacobs et al., 2020), suggesting that the pregnancy-specific bodily and emotional state may shape how guilt is sustained in these nurses’ experience.

We also found that prolonged self-blame substantially interfered with psychological detachment. Detachment—the ability to mentally disengage from work during rest—is considered a key recovery mechanism, helping to relieve negative emotions and restore psychological resources (Sonnentag and Fritz, 2015). Yet, pregnant nurses in this study struggled to detach due to enduring guilt, leading to a cycle of negative emotions. This supports the theoretical premise that high-intensity emotional stressors (such as pediatric death) can override standard detachment strategies, requiring more than just time off to resolve. This contrasts with the findings that most emergency nurses can gain some relief through social and leisure activities during non-work hours (Lee and Ashforth, 1996). Our findings therefore suggest that pregnant nurses are particularly vulnerable in their ability to achieve psychological detachment and require targeted external interventions.

### Protective role of emotional support systems

Social and emotional support demonstrated significant protective effects in this study. Nurses who received care from family and colleagues showed gradual decreases in anxiety and faster recovery. This is consistent with Jacobs et al., who emphasized the importance of social support for emotional recovery among healthcare workers (Baskin and Bartlett, 2021). However, unlike Jacobs’ finding that support benefits all nurses equally, our study observed that late-pregnancy nurses, despite receiving support, still exhibited slower recovery. This suggests that in late pregnancy, reliance on family and peer support alone is insufficient, highlighting the need for professional psychological intervention and multidisciplinary collaboration.

Our findings also suggest that the effectiveness of support varied by source. Emotional support from family primarily alleviated fetal health-related concerns, while professional support from colleagues helped reduce work-related guilt. This aligns with Huang et al., who emphasized the value of diverse support networks in fostering resilience among healthcare professionals (Jacobs and Dodd-Butera, 2018). Therefore, for pregnant nurses experiencing major traumatic events, it is crucial to establish multi-layered support systems encompassing family, peers, and organizational resources.

### Interaction of physiological and psychological factors

Another key finding of this study is the complex interaction between physiological and psychological factors in shaping recovery trajectories. Hormonal fluctuations during pregnancy have been described as challenging emotional regulation capacity, while heightened concerns about fetal health further increase psychological burden (Zhang et al., 2022). In our study, late-pregnancy nurses experienced these combined bodily and emotional pressures as making it particularly difficult to regain emotional balance, which helps to explain why they described the slowest recovery. Unlike Zhang et al., who studied general pregnant workers (Clayborne et al., 2022), our sample consisted of emergency nurses, whose high-intensity work may have amplified how these interactions were felt and interpreted. At the same time, our study observed the positive turning point associated with childbirth. Some late-pregnancy nurses reported marked reductions in anxiety and gradual emotional stabilization postpartum. This corresponds with Marigold et al. (2023), who found that postpartum mother–infant interaction plays a critical role in emotional recovery. Thus, pregnancy and postpartum stages may exert distinct effects: the former is often lived as a period of heightened vulnerability, while the latter may facilitate recovery through new forms of embodied connection and meaning.

### Influence of educational background on the lived experience

An additional factor that appeared to shape variation in the lived experience was the participants’ educational attainment. Differences in education level and professional maturity influenced how nurses articulated and interpreted emotionally distressing events. Participants with higher educational backgrounds tended to provide more reflective, structured, and interpretive narratives, whereas those with lower educational levels often described their experiences in more immediate, affect-laden terms. These patterns resonate with recent empirical work showing that educational level is associated with differences in nurses’ emotional competencies and mental health indicators (Wang P. et al., 2023). Similar findings have been reported among nursing undergraduates, where greater educational exposure was linked to more developed emotional management and communicative competence in stressful situations (Oliveira Silva et al., 2021). Educational interventions have also been shown to improve nurses’ emotional intelligence (Wang A. et al., 2023), further suggesting that educational background and training shape how emotional experiences are processed and expressed in clinical settings. While these differences did not alter the core phenomenological themes identified in this study, they contributed to variations in narrative richness and should be considered when interpreting the findings.

### Limitations and future directions

Several limitations should be noted. First, the three-month follow-up period was relatively short, preventing the full exploration of long-term trajectories across the postpartum transition. Second, this study was observational and did not evaluate the effectiveness of specific interventions. Third, the study was conducted in a single tertiary hospital with a small sample size (*n* = 18) determined by data saturation principles. While sufficient for phenomenological depth, the descriptive quantitative findings should be interpreted with caution and may not be generalizable to vary cultural contexts. Fourth, while we explored the intersection of pregnancy and grief, future research should adopt biopsychosocial or critical–interpretive paradigms to gain a more holistic understanding of this phenomenon. It is essential to investigate how structural realities—such as night-shift policies, staffing shortages, and professional hierarchies—interact with biological vulnerabilities and social networks to shape nurses’ experiences. Integrating these broad frameworks will help move research beyond individual-level analysis to address the systemic and organizational determinants of professional wellbeing.

## Conclusion

This longitudinal study reveals that the emotional recovery of pregnant emergency nurses following pediatric deaths is not solely a psychological process but is heavily constrained by structural and biological realities. While early- and mid-pregnancy nurses generally achieved recovery, those in late pregnancy and night-shift roles faced prolonged distress due to the cumulative burden of physiological vulnerability and workplace isolation. Our findings indicate that impediments to recovery—such as guilt and impaired detachment—are exacerbated by systemic factors, including night-shift arrangements, variations in professional training, and the accessibility of social support networks. Therefore, effective support must extend beyond individual psychological counseling to include structural organizational reforms, such as optimizing shift schedules for high-risk groups and strengthening professional support systems, to foster resilience and patient safety.

## Data Availability

The raw data supporting the conclusions of this article will be made available by the authors, without undue reservation.
